# Supplementary figures and images for: A new species of Gulo from the Early Pliocene Gray Fossil Site (Eastern United States); rethinking the evolution of wolverines
Source: PeerJ. 2018 Apr 18;6:e4648. doi: 10.7717/peerj.4648 (PMC5910791; doi:10.7717/peerj.4648)

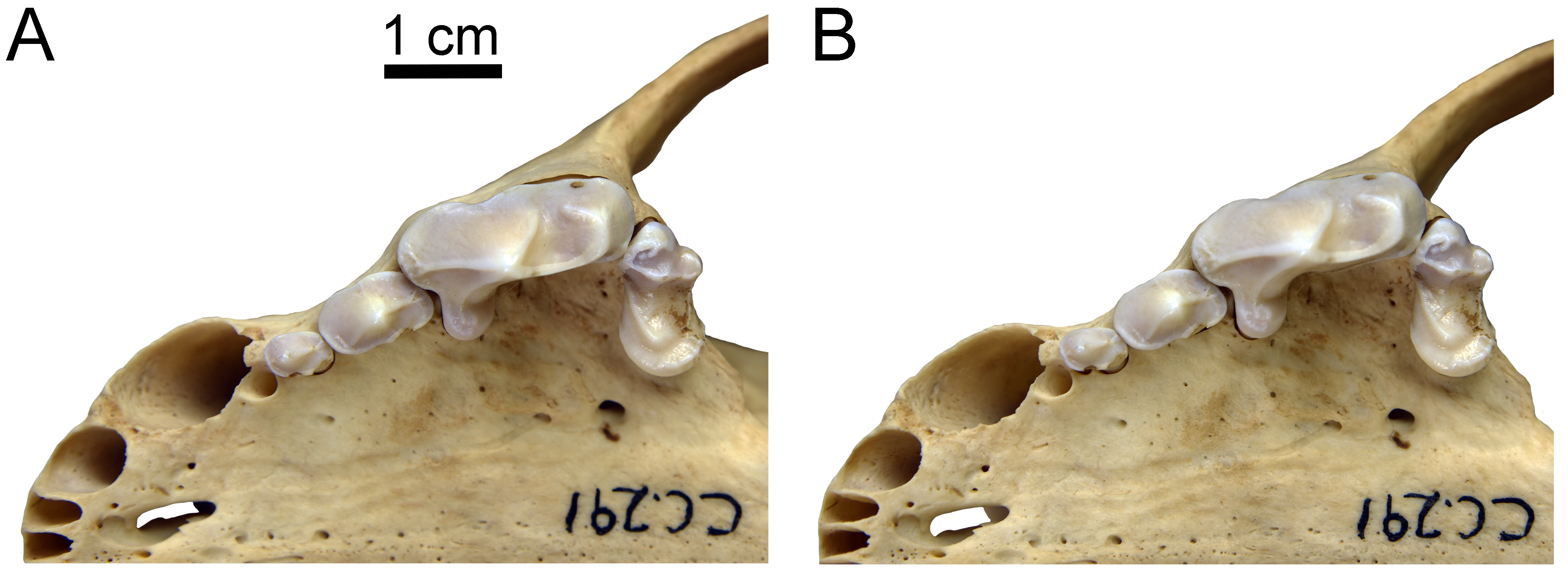

Supplement: Figure S1 — (A) Palate parallel to the photographic plane. (B) Alveolar margin of P4 parallel to the photographic plane. Note that in A the lingual cingulum along the P4 metastyle is obscured, but it is visible in B. Scale bar equals 1 cm. Photographs by Joshua Samuels. [file peerj-06-4648-s001.png]
